# Excimer laser 6^th^ generation: state of the art and refractive surgical outcomes

**DOI:** 10.1186/s40662-015-0015-5

**Published:** 2015-03-01

**Authors:** Mohamed El Bahrawy, Jorge L Alió

**Affiliations:** Vissum Corporación Alicante, Alicante, Spain; Division of Ophthalmology, Universidad Miguel Hernández, Alicante, Spain; Clinical research fellow in Vissum Corporación Alicante, Universidad Miguel Hernández, Alicante, Spain; Avda de Denia s/n, Edificio Vissum, 03016 Alicante, Spain

**Keywords:** Refractive surgery, Excimer laser, 6^th^ Generation

## Abstract

After nearly three decades of innovation in excimer laser, today we are presented with a state of the art generation targeting minimally invasive refractive surgery with high speed laser, faster trackers, pupil monitoring systems and better customization profiles. These systems are capable of delivering better treatments with less induced postoperative high order aberrations. The results reported by many authors had confirmed the superiority in efficiency and safety profiles of this generation compared to previous generations. Still, current technology is facing major challenges in the correction of high hyperopic errors and in presbyopic treatments, with upgrades in ablation centration and thermal control needed, which will ensure better biomechanical results, as a step closer to perfection in refractive surgery.

## Introduction

### The birth and evolution of excimer laser refractive surgery

The concepts of modern refractive surgery witnessed its breakthrough when Professor Jose I. Barraquer described in 1949 his coined technique of *keratomeliosis,* setting the foundation for all following innovation in this field. The name *excimer laser* came as an abbreviation of “*excited dimer*”, introduced by the Russian, Nikolay Basov, in 1970 using a xenon dimer gas [1]. Few years later, the argon-fluoride excimer laser was developed and was first tried on an organic tissue by IBM scientists. The introduction of excimer laser to be used in the human eye was done by Stephen Trokel as a precise and safe tool of corneal shaping*,* these concepts later defined the refractive techniques widely used now, when Marguerite McDonald under the supervision of Steve Kaufmann, performed the most commonly used epithelium removal technique *photorefractive keratectomy* (PRK). Peyman, presented the first patency of using excimer laser as a corneal refractive tool, and it was accepted in Jun 1989 (personal correspondence Gholam Peyman). Following Ioannis Pallikaris, among others, introduced the most widely used and commonly accepted technique of *laser in situ keratomeliosis* (LASIK) in 1990 [1].

Several ophthalmic authorities had set the benchmark for laser keratorefractive surgery; The Food and Drug Administration (FDA) based on data presented by several evidence based reviews, defined the correction limitation of excimer laser (Table 1) [2]. The American Academy of Ophthalmologist (AAO) reports stated that the substantial level II and III evidence proved that excimer laser refractive surgery whether LASIK or PRK, is a safe and effective tool of correcting the full spectrum of refractive errors but with some limitations in high hyperopic refractive errors [2,3].

**Table 1:** **FDA Indications for LASIK and PRK:** [2]

	**LASIK**	**PRK**
**Myopia**	Less than −14.0 D with or without astigmatism between −0.50 and −5.00 D	Up to −12.0 D with or without astigmatism up to −4.00 D
**Hyperopia**	Up to + 5.00 D with or without astigmatism up to +3.00 D	Up to +5.00 D with or without astigmatism up to +4.00 D
**Mixed astigmatism**	Astigmatism up to 6.00 D, cylinder is greater than sphere and of opposite sign.	

[2] AAO Refractive Management/Intervention PPP Panel, Hoskins Center for Quality Eye Care. Refractive Errors & Refractive Surgery PPP – 2013. 2013. Retrieved (May 8, 2014) from: http://one.aao.org/preferred-practice-pattern/refractive-errors—surgery-ppp-2013.

The previous lasers were presented with a number of limitations, where treatment of hyperopia and to a greater extent presbyopia, represented the fundamental challenge as the biomorphological and biomechanical corneal structure and architecture seem to render the planned correction [4]. Early broad beam lasers were associated with Laser-induced deviations from the intended uniform corneal profiles, increasing depth ablation and therefore decreased the predictability of the refractive outcomes [5]. The variations in energy of the laser beam that could happen during a refractive surgical procedure also reduced refractive predictability, fluctuations noted between two series of pulses averaged 11.02%, tending to decrease progressively till the end of the treatment and the total loss of energy was 45.16% [6]. Another major limitation is biological interactions, as wound healing responses are thought to be a key factor limiting the predictability of refractive surgery in some patients and may contribute to some of the complications, including haze formation [7]. Also a central island is a type of astigmatism that occurs after laser refractive surgery. It is generally defined as a central area of steeper corneal tissue having increased refractive power [8]. This evolutional technology that passed by several generations demonstrated in (Table 2).

**Table 2:** **Features of the successive generations of Excimer Lasers**

1st Generation:	Pre-clinical (Touton, VISX, Summit)
2nd Generation:	Broad beam laser, fixed optical zone
3rd Generation:	Broad beam laser, variable optical zone, multizone treatment
4th Generation:	Flying spot laser, built in tracker, hyperopic treatment
5th Generation:	Customised wavefront (guided, optimised) treatments
6^th^ Generation:	• Faster ablation rates & tracking systems
	• Lower biological interaction
	• More variables under control
	• Pupil size
	• Advanced ablation profiles
	• Ciclotorsion control
	• Online pachymetry

Original Table.

## Review

### Sixth generation excimer lasers

**Figure 1:**
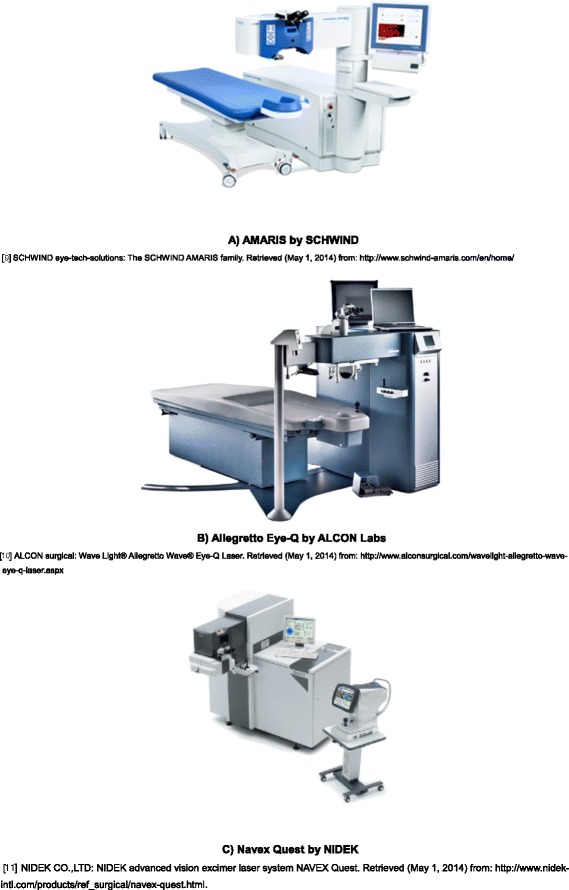
**Three 6** ^**th**^ **generation excimer laser platforms.**

This generation of excimer laser platforms can be defined as an excimer laser delivery system that targets the goal of minimally invasive laser refractive surgery by reducing the amount of time and tissue ablated with a faster laser system, delivering more laser spots per second, with a faster treatment time, through the ability of ablating more corneal tissue in a given time (Table 3) [9-11]. The 6^th^ generation lasers speed varies from 400 to 1050 Hz, being 400 Hz in Wavelight Eye-Q up to 1050 Hz in Schwind Amaris. On average, a 500 Hz platform will reduce the time needed per diopter ablation in a 6.5 mm optical zone from 7–10 seconds using older generation laser platforms to an effective 4 seconds [12]. Another feature to reduce treatment time is the advanced fluence level adjustment system, in which a mix of high and low fluence levels are used. High fluence level will perform 80% of corneal ablation, while low fluence will be used for fine correction, improving resolution, with remarkable precision in high refractive errors (Figure 1).

**Table 3:** **Comparison between 6** ^**th**^ **generation Excimer platforms**

**Company**	**Schwind**	**Nidek**	**WaveLight**
**Model**	**Amaris**	**Navex Quest**	**AllegrettoEye-Q**
**Laser type**	ArF	ArF	ArF
**Laser beam**	Flying Spot	Slit scanning + variable spot-size scanner	Flying spot
**Beam profile**	Super-Gaussian	Flat Top	Gaussian
**Pulse rate**	500-700 Hz	6 scans/sec.60 Hz max	400-700 HZ
**Pulse duration**	10 ns	25 ns	10 ns
**Peak fluence**	160 mJ/cm^2^ - 450 mJ/cm^2^	130 mJ/cm^2^	400 mJ/cm^2^
**Beam size**	0.54 mm	10 x2 mm scanning slit (1 mm for customized and hyperopia)	0.68 mm
**Spot size (cornea)**	0.54 mm	1.0 mm	0.95 mm (1.2 mm)
**Optical zone (OZ)**	4 - 10 mm	6.5 mm	4.5 mm - 8 mm
**Ablation zone**	Optimized	8 mm	9 mm
**Ablation profile**	Aspheric (aberration free)	Munnerlyn with aspheric tansition zone	Aspheric (including Q-value)
**Transition zones adjustable**	No	Yes	Yes
**Static Cyclo-Torsion**	Yes	Yes	Pseudo, yes
**Dynamic Cyclo-Torsion**	Yes	Yes (TEC = torsion error correction)	Pseudo, yes
**Cyclo-Torsion, Sampling Rate**	36 Hz	30 Hz	NR
**Ablation depth per shot (cornea)**	0.42 μm - 0.68 μm	0.32 μm	0.65 μm
**Ablation volume per shot (cornea)**	110 pl -220 pl	250 pl	N/A
**Ablation depth per diopter (6.5 mm OZ)**	16.4 μm	15 μm	15.3 μm
**Time per diopter (6.5 mm OZ)**	<2.5 ms	5 s	3 s
**Ablation depth -5dpt/OZ = 6 mm**	65 μm (12 s)	63 μm	65 μm
**Eye tracking system**	Active video tracking (SMI)	Active video tracking (SMI)	Active video tracking (SMI)
**Sampling rate**	1050 Hz	1050 Hz	400 Hz
**Eye tracker response time**	<3 ms	4 ms	4 ms
**Cyclo-Torsion, Resolution**	Static ±15° Dynamic ± 7°	NR	NR
**X-Y & Z-tracking**	Active	Active	No
**Presbyopic treatment**	No	Yes	No
**Online pachymetry**	Yes - integrated	No	No
**Eye fixation**	Green LED	Yes	LED
**Centration of pupil**	Automatic, user defined	Manual	User defined
**Laserhead/Lasersource**	Coherent	Lambda	TUI
**FluenceTest needed every:**	2 hours	NR	Before every treatment day
**Fluence & Calibration**	Automatic and objective	Manual and subjective	Manual and subjective
**Capable of customized ablation**	Yes	Yes	Yes
**Ocular wavefront**	Yes	Yes	Yes
**Method used for wavefront**	Hartmann - Shack	Yes	Tscherning Principle
**Topographic system**	Corneal Wavefront Analyzer/CSO	Topographer retinoscopy	Yes Oculus
**Topgraphic link**	Yes (Corneal wavefront)	Yes (OPD-scan)	Yes (topographic based on Zernike)
**Dimensions (LxWxH)**	264 × 144 × 136 cm(including patient bed)	137 × 151.6 × 147 cm	120 × 145 × 130 cm (without patient bed)
**Weight (without patient bed)**	550 kg	650 kg	265 kg (without bed and gas) patient bed 188 kg
**Website: last visited May 2014**	http://www.schwind-amaris.com/en/home/	http://www.nidek-intl.com/products/ref_surgical/navex-quest.html	http://www.alconsurgical.com/wavelight-allegretto-wave-eye-q-laser.aspx

[9] SCHWIND eye-tech-solutions: The SCHWIND AMARIS family. Retrieved (May 1, 2014) from: http://www.schwind-amaris.com/en/home/.

[10] ALCON surgical: Wave Light® Allegretto Wave® Eye-Q Laser. Retrieved (May 1, 2014) from: http://www.alconsurgical.com/wavelight-allegretto-wave-eye-q-laser.aspx.

[11] NIDEK CO., LTD: NIDEK advanced vision excimer laser system NAVEX Quest. Retrieved (May 1, 2014) from: http://www.nidek-intl.com/products/ref_surgical/navex-quest.html.

The reduction of induced aberrations, is a critical trend in modern laser refractive surgery, 6^th^ generation platforms feature advanced ablation profiles, with the reduction of spot size as a key factor of control of the induced aberration; which is 0.54 mm and 0.68 mm for Schwind Amaris and Allegretto Eye-Q respectively, compared to a spot size of 0.8 mm or more for conventional excimer lasers. Also, these profiles are able to correct pre-existing optical aberration through integrated customized and wavefront ablation technology. The efficiency of the previous features requires extremely accurate laser spot placement, in which the eye tracker latency time is of only 1.6 milliseconds (ms). A conventional laser platform eye tracker will have a capturing rate of 60 to 330 Hz, able to detect the pupil position at 4000 Hz, with a response time of 36 ms, clearly not fast enough for a high speed laser platform of a speed reaching 700 Hz. The new five dimensional turbo speed tracker have an acquisition speed of 1050 Hz generating a response rate less than 3 ms with unique rotational balance, tracking both the pupil and the limbus [13].

A conventional eye tracker adjusts eye movements into an X and Y-axis linear movement, and lasers are able to follow eye rolling*,* through translation of these linear movements into rotations with the help of an eye model, so that these generated rotations can be followed and compensated. Modern eye trackers do not only follow these horizontal and vertical displacements of the eye but also track the cyclotorsional rotations. These cyclorotations can be classified into static cyclorotation component (SCC); occurs when the patients move from an upright to a supine position and a dynamic cyclorotation component (DCC); which occurs during the treatment procedure [14].

The high repetition rate of excimer lasers may result in shorter interval between laser pulses on the same area of the cornea, increasing the thermal load on the cornea and resulting in thermal damage, as a safety feature, a recent generation laser will use an intelligent thermal effect control to significantly reduce the heating effect on the cornea, the area around the applied laser spot is blocked for a certain time allowing the cornea to cool down, this will generate a dynamic thermally optimized distribution of the laser pulses, with enough time for each spot to cool down between pulses. The thermal load from refractive corrections using a 500 Hz laser with a fluence of 500 mJ/cm^2^ was recorded with an infrared thermography camera to analyze and evaluate each single *in vivo* measurement; the overall maximum temperature change induced by the refractive ablations was ≤4°C, the increase in the peak temperature of the ocular surface never exceeded 35°C, and this low thermal load was independent of the amount of correction the eye achieved [15].

Another safety feature is the automatic monitoring of the pupil size, as illumination is automatically adjusted in such a way that the pupil is exactly the same size at the start of the treatment as it was on the preoperative examination. Finally, the integrated online pachymetry in these state of the art platforms will display the corneal thickness in real time, with the ability to monitor the targeted measurements before and after flap lifting as well as during and after laser ablation, which is documented in the treatment log at the end of the procedure [9-11].

#### Outcomes of 6^th^ generation lasers

Our studies focused on the promised safety and accuracy of the new platforms (Table 4) [13,14,16-23] in myopic correction through two separate reports of patients with high myopia of −8.50 D or more. In the first study, we studied 29 eyes of 17 patients, with a mean age of 36.65 ± 10.80 years, and a mean spherical equivalent (MSE) of −8.39 ± 0.93 D, after 6 months follow up. We found the efficacy of the treatment to be 89.6% within ±1.00 D of target refraction, and post operative high order aberrations (HOA) to be 0.95 ± 0.80 μm [13]. We confirmed the results with a larger sample in a second study, to show an efficacy of 84.3% within ±0.5 D of target refraction [18]. At the same time, Arba-Mosquera et al. published results of the 3 month follow up of 30 eyes with a mean spherical equivalent (MSE) of −4.27 ± 1.62 D showing a mean residual spherical equivalent of −0.07 ± 0.25 D and postoperative HOA of 0.425 ± 0.129 μm [14]. Later on, Tomita et al. used a larger sample of 10235 eyes of 5191 patients. In this study, the MSE was −5.02 ± 2.17 D and follow up results up to 3 months showed an efficacy index of 1.00; 88.4% within ±0.50 D of target refraction and a safety index of 1.03; 96.9% achived 0.0 LogMAR or better, and postoperative HOA were 0.70 ± 0.23 μm [17]. The previous studies were conducted on the AMARIS excimer laser platform of SCHWIND eye-tech-solutions, Kleinostheim, Germany. Kanellopoulos et al. reported his results with the Alcon-WaveLight EX500 excimer laser by (Alcon Laboratories, Fort Worth, TX), where he followed 58 patients who underwent bilateral surgery for 12 months. The preoperative MSE was −7.67 ± 1.55 D, and his results showed 100% were within 1.0 D defocus equivalent with a keratometric stability of 0.22 D [24].

**Table 4:** **Outcomes of the AMARIS-SCHWIND 6** ^**th**^ **generation excimer laser**

**Author**	**Number of patients**	**Number of eyes**	**Mean age (years)**	**Gender (female/male)**	**MSE (D)**	**HOA (μm)**	**Efficacy**	**Efficacy index**	**Safety index**	**Fellow up (months)**
**Myopic Patients**										
Tomita et al. [16]	685	1280	34 ± 8 (18–65)	371/314	−4.89 ± 2.12 (−0.5 to −11.63)	0.66 ± 0.20	96.6 % 20/20	1.02	1.06	3
							94.1% ±0.5 D			
Tomita et al. [17]	5191	10235	33.9 ± 7.89	2428/2763	−5.02 ± 2.17 (−2.75 to 11.50)	0.70 ± 0.23	96.9% 0.0 LogMAR	1	1.03	3
							88.4% ±0.5 D			
Vega-Estrada et al. [13]	17	29	36.65 ± 10.80	N/A	−8.39 ± 0.93	0.95 ± 0.8	89.6% ±1.00 D	NR	Not reported	6
							0.11 ± 0.26 LogMAR			
Alió et al. [18]	32	51	23-61	N/A	≥ − 8.5	NR	84.3% ±0.5 D	NR	Not reported	6
Arba-Mosquera et al. [14]	NR	30	33 (19–49)	53/47	−4.27 ± 1.62 (−7.38 to −1.38)	0.425 ± 0.129 (P < 0.01)	0.47 ± 0.72 lines (P < 0.05)	NR	Not reported	3
							−0.07 ± 0.25 (−0.63 to +0.50)			
**Hyperopic Patients**										
Alió et al. [19]	28	51	Not reported	NR	+5.64 ± 0.93 (3.50 to 7.88)	−0.44 ± 0.22 (P = 0.00)	70.37% ±0.5 D	0.85	0.94	6
Arbelaez et al. [20]	50	100	37 (21–59)	54% Females	+3.02 ± 2.06 (+0.13 to +5.00) +1.36 ± 1.61 (Ast.) (0.00 to 5.00)	↑ 0.18 (P < 0.05)	90% 20/20	0.89	1.1	6
							89% ±0.5 D			
							94% ±0.5 (Ast.)			
**Astigmatic Patients**										
Alió et al. [21]	36	52	21-53	NR	Mixed Ast. > 3.0	NR	65.3% ±1.0 D	NR	Not reported	3
Arbelaez et al. [22]	200	360	NR	NR	−0.14 ± 0.31	↑ 0.09	97% ±0.5 D	NR	65% No changes	6
					+0.25 ± 0.37 (Ast.)					
Arbelaez et al. [23]	NR	358	NR	NR	−3.13 ± 1.58	↑ 0.09	98% 20/20	NR	Not reported	6
					−0.69 ± 0.67 (Ast.)		96% ±0.5 D			

MSE: mean spherical equivalent, HOA: high order aberrations, Ast.: astigmatism, NR: not reported, N/A: not available.

↑: increase in high order aberrations.

The small spot *hyperopic laser in situ keratomileusis* (H-LASIK) ablation at the periphery of the cornea producing certain degrees of steepness. This treatment modality had several limitations as decentration, decrease in best corrected visual acuity (BCVA), high frequency for the need of retreatments, residual refractive errors and induction of astigmatism due to the high levels of corneal aberrations as negative spherical aberration, all cause loss of the efficiency of treatments and changes in biomechanics of the cornea [20]. Our studies included a sample of 51 eyes in 28 patients of a MSE of +5.64 ± 0.93 D followed up for 6 months, we reported an efficacy of 70.37% within ±0.5 D of target refraction, the HOA postoperatively was −0.44 ± 0.22 μm, with a efficacy and safety index of 0.85 and 0.94 respectively [19]. Another 6 months retrospective follow up of 51 eyes with a spherical equivalent of more than 5.5 D showed significant increase in corneal root mean square (RMS) HOA, RMS spherical aberration (SA) and RMS coma were also observed six months after surgery (p < 0.01). Corneal asphericity for the 4.5 mm (Q45) and 8 mm (Q8) of corneal diameter also changed significantly during the postoperative period (p < 0.01). Strehl ratio change was not statistically significant (p = 0.77) [25]. Arbelaez et al. published a study with MSE +3.02 ± 2.06 D (astigmatism was +1.36 ± 1.61 D), they reported 6 months follow up with a mean postoperative increase in HOA of 0.18 μm (P < 0.05), 89% were within ±0.5 D of the target refraction and 94% were ±0.5 D of target astigmatism [20]. Kanellopoulos used a larger sample of 202 eyes with a longer follow up of 2 years to demonstrate the safety and efficacy of topography guided ablation using a 400 Hz WaveLight excimer laser by (Alcon Laboratories, Fort Worth, TX). In his study the MSE was +3.04 ± 1.75 D, the results showed that the mean refraction spherical equivalent was ±0.50 D of target refraction in over 80% of cases, with an increase in the RMS of 15% [26].

Patients’ satisfaction after refractive surgery, wavefront guided or not, is primarily dependent on the successful treatment of lower order aberrations of the sphere and cylinder of the eye. LASIK has been successful in the correction of mild to moderate myopic astigmatism, but with limited reports on the efficacy, predictability and safety of it in higher myopic astigmatism in the terms of astigmatic correction of HOA, with limitation of retreatments needed [12]. We reported the 3 months follow up of 52 eyes of mixed astigmatism of more than 3 D, in which the efficacy was 65.3% within ±1.0 D of target refraction [21]. Earlier, Arbelaez et al. published a number of reports, with an average of 1.26 ± 3.29 D of astigmatism, and with 6 months follow up found an increase of 0.09 μm with a residual astigmatism 0.50 ± 0.26 D (P < 0.0001) [12,22,23]. The mean decrease of astigmatic magnitude in these reports was 93%, indicating a slight under correction of the preoperative astigmatism, but with marked improvements from the 36% to 91% reported with the use of older excimer laser platforms [27,28]. Also, recent reports showed a 100% efficacy within ±0.25 D after 12 months [24]. Topography-guided hyperopic astigmatism correction showed a correction of a mean preoperative cylinder value of −1.24 ± 1.41 D to the respective postoperative value of −0.35 ± 0.25 D [26].

New platforms had shown similar satisfactory results with PRK. Aslanides et al. in his 2 years follow up of 80 eyes with mild to high myopia and myopic astigmatism found 91% to be within ±0.5 D of target refraction, but reported a statistically significant increase in postoperative coma (+0.12 μm) and spherical aberration (+0.14 μm) compared to preoperative values (*P* < 0.001, both cases) when performed using the SCHWIND AMARIS [29,30].

### Challenges facing excimer laser refractive surgery today

In spite of the astonishing progress in the field of laser refractive surgery, a number of challenges are still facing the technology. One of the most critical is the limitations in the visual and optical outcomes in patients with high hyperopic refractive errors, as most authors reported a significant induction of the corneal HOA, most significantly in the RMS coma [19,20].

The efficacy of the excimer laser technology even with latest platforms in the treatment of presbyopia is still the subject of great debate. PresbyLASIK has been described in three different approaches to create multifocality: the transitional multifocality, the central presbyLASIK and the peripheral presbyLASIK. Both central and peripheral techniques reportedly obtained adequate spectacle independence for near and for far with a degree of neuroadaptation process needed in peripheral techniques. An intentional increase in coma aberrations was noticed in transitional multifocality giving it a range of very limited use and outcomes. The level of scientific evidence we already have is enough to consider presbyLASIK as a useful tool in the correction of presbyopia [31]. Epstein et al. reported a 4 years follow up of 103 patients who underwent peripheral presbyLASIK, 89% of hyperopes and 92% of myopes was completely spectacle independent, with distance unaided visual acuity of 20/20 in 67.9% of hyperopes and 70.7% in myopes. They also reported that the overall increase in the HOA was manifested to a greater extent in hyperopic cases [32]. Other programs as The Supracor presbyopia procedure showed good near visual acuity outcomes over 6 months follow up, but loss of corrected distance visual acuity (CDVA) occurred in 39.1% of eyes, also reported in PresbyMAX [33]. At 1 year, 70% of patients achieved uncorrected distance visual acuity (UDVA) 0.1 logMAR or better, 84% patients obtained uncorrected near visual acuity (UNVA) 0.1 logRAD or better, and 83% of eyes were within 0.75 D of defocus [34].

Ablation centration is a major issue in the excimer laser development, the decentration of ablation can lead to under correction and irregular astigmatism, which is most important in hyperopic patients [35,36], who tend to have a larger angle kappa values [35]. There are four main methods of centration in laser refractive surgery that has been suggested in literature; center of the pupil, coaxially sighted corneal light reflex (CSCLR), corneal vertex normal and between the pupillary and visual axis [37]. Many reports had demonstrated that pupil-centered and vertex-centered treatments provide similar visual and optical outcomes. However, in eyes showing large temporal pupil decentration, pupil-centered ablation seemed to produce a lower amount of coma and consequently, a reduced loss of BCVA compared with vertex-centered patients [26,38,39].

One of the other challenges is the limitation facing wavefront-customized treatment. Despite decreases in spot size of up to 0.5 mm and thermal damage control limitations still exist, mainly through the biomechanical changes induced by the wound healing patterns [40]. It was reported that one month after treatment, corneal hysteresis and the corneal resistance factor decreased significantly from 10.44 to 9.3 mmHg and from 10.07 to 8.13 mm Hg, respectively [41]. So, when looking to customization as the planning of the most optimum ablation pattern specifically for each individual eye based on its diagnosis and visual demands, the best approach is a sophisticated pattern [22]. As wavefront guided treatment may increase the HOA up to 100%, as the induction related to the baseline levels of HOAs, is more significant in patients with less than 0.3 μm or greater than 0.3 μm of HOA. But results of this customized treatment still showed promising results as Arbelaez et al. reported an average change in coma from 0.38 μm to 0.31 μm (a 19% decrease) (P = 0.04), in trefoil from 0.35 μm to 0.12 μm (a 66% decrease) (P = 0.0005), and in spherical aberration from +0.14 μm to +0.08 μm (a 48% decrease) (P = 0.02) [22]. Furthermore, when comparing a small spot scanning laser and a variable spot scanning laser, Yu et al. reported in his study of 50 patients, in which one eye of each patient was treated by the small spot laser using Allegretto Wave Eye-Q system and the other with the variable-spot scanning laser of Visx Star CustomVue S4 IR system; the small-spot scanning laser group had significantly less spherical aberration (0.12 versus 0.15) and significantly less mean total higher-order RMS (0.33 μm versus 0.40 μm) [42].

Patient satisfaction with the results of treatment with the recent excimer laser was remarkable. Kyprianou et al. reported a 100% satisfaction in 32 patients, with average age of 31.9 years and a preoperative MSE of −3.05 D. This was evaluated by a questionnaire consisting of 21 questions. Patients were most satisfied in questions concerning quality of vision, distance vision, when watching TV and driving during daytime and during the night [43].

## Conclusion

In summary, the latest generation of excimer laser platforms had introduced a large number of features as faster laser, smaller spot size, a high speed tracker, pupil monitoring and online pachymetry, all of which provided superior treatment with significant improvement of induced post operative HOA and control of thermal damage. This technology is still facing major limitations in terms of high hyperopic, presbyopic treatments, along with difficulties in laser centration along with the limitation of the customized treatments, generated by the biomechanical patterns of wound healing [44].
